# Warm Autoimmune Hemolytic Anemia in an Elderly Patient With a History of Chronic Idiopathic Thrombocytopenic Purpura: A Case Report

**DOI:** 10.7759/cureus.108289

**Published:** 2026-05-05

**Authors:** Ariana T Pitaro, Shilpa Bhat, Muhammad Z Ali, Alainna Cavin, Reshma K Abraham

**Affiliations:** 1 Internal Medicine, Kent Hospital, University of New England College of Osteopathic Medicine, Portland, USA; 2 Internal Medicine, Kent Hospital, Brown University, Warwick, USA

**Keywords:** autoimmune hemolytic anemia (aiha), evans syndrome, igg immunoglobulin, itp in adult, warm autoimmune hemolytic anemia

## Abstract

Warm autoimmune hemolytic anemia (AIHA) is a rare immune-mediated disorder characterized by IgG-mediated hemolysis and laboratory evidence of anemia, reticulocytosis, elevated lactate dehydrogenase (LDH), indirect hyperbilirubinemia, decreased haptoglobin, and a positive direct antiglobulin test (DAT). In elderly patients, diminished bone marrow reserve may limit compensatory erythropoiesis, resulting in more severe presentations.

We report the case of an 82-year-old man with a history of chronic idiopathic thrombocytopenic purpura (ITP) who presented with profound anemia without overt bleeding. Laboratory findings were consistent with warm AIHA. He required multiple packed red blood cell (pRBC) transfusions and initiation of high-dose corticosteroids, with subsequent hematologic improvement. His course was complicated by new-onset atrial flutter, managed with rate control, as anticoagulation was deferred due to severe anemia and bleeding risk. Prior evaluation, including bone marrow biopsy, excluded malignancy.

This case highlights the diagnostic and therapeutic challenges of warm AIHA in elderly patients with prior autoimmune cytopenias. The sequential occurrence of ITP and AIHA raises concern for Evans syndrome and reflects underlying immune dysregulation. This case was further complicated by new-onset atrial flutter, creating a management dilemma regarding anticoagulation in the setting of severe anemia and bleeding risk. Management requires careful coordination to balance hemolysis, comorbidities, and competing thrombotic and hemorrhagic risks.

## Introduction

Autoimmune hemolytic anemia (AIHA) is a rare immune-mediated hematologic disorder characterized by premature destruction of red blood cells secondary to autoantibody formation against erythrocyte surface antigens [1].

AIHA is rare, with an estimated incidence of approximately 1-3 cases per 100,000 persons per year [1]. In elderly patients, diagnosis and management may be more complex due to reduced bone marrow reserve, impaired erythropoietin responsiveness, immune senescence, and higher rates of comorbid disease [2]. This case is notable because warm AIHA occurred in the setting of prior chronic ITP, raising concern for Evans syndrome, and was further complicated by new-onset atrial flutter.

Warm AIHA, the most common subtype, is mediated primarily by IgG antibodies active at body temperature and results in Fc receptor-mediated extravascular hemolysis within the spleen [1,2]. Patients typically present with a clinical picture that includes anemia, reticulocytosis, a positive direct antiglobulin test (DAT), elevated lactate dehydrogenase (LDH), indirect hyperbilirubinemia, and decreased haptoglobin [1,3-5]. In elderly populations, age-related immune senescence, reduced bone marrow reserve, and impaired erythropoietin responsiveness may blunt compensatory erythropoiesis, resulting in more profound or symptomatic anemia [6]. Diagnosis is often complicated by competing etiologies of anemia, including chronic disease, nutritional deficiencies, renal insufficiency, and malignancy [5,6].

The presence of prior autoimmune cytopenias, such as idiopathic thrombocytopenic purpura (ITP), raises concern for Evans syndrome, defined as the sequential or concomitant occurrence of two or more autoimmune cytopenias [7]. Such cases reflect underlying dysregulation of immune tolerance and may carry a more complex clinical course. This case highlights the clinical complexity of diagnosing and managing warm AIHA in elderly patients with prior autoimmune cytopenias and multiple comorbidities.

## Case presentation

An 82-year-old man with a past medical history of chronic ITP, previously treated with rituximab, intravenous immunoglobulin (IVIG), and corticosteroids, with the last rituximab course administered eight years prior, presented with profound acute-on-chronic anemia without overt bleeding. His medical history was notable for prostate cancer treated with brachytherapy six years prior, hypertension, type 2 diabetes mellitus, and hyperlipidemia. Four weeks prior to admission, he required transfusion of four units of packed red blood cells (pRBCs) for hemoglobin below 7 g/dL. At that time, a full hemolysis evaluation was not completed, and the patient was not formally assessed for AIHA prior to this admission. Therefore, it is unclear whether this earlier episode represented evolving AIHA, multifactorial anemia, or an earlier manifestation of Evans syndrome.

Given his history of malignancy and worsening cytopenia, an outpatient bone marrow biopsy was performed two weeks prior to admission and demonstrated no evidence of marrow infiltration or hematologic malignancy, supporting a nonmalignant etiology. Given his history of prostate cancer, a malignancy-associated or paraneoplastic process was considered; however, bone marrow biopsy did not show marrow infiltration or hematologic malignancy.

On examination, the patient appeared cachectic and jaundiced with scleral icterus. Laboratory studies demonstrated severe hemolytic anemia with elevated hemolysis markers, reticulocytosis, hyperbilirubinemia, positive IgG DAT, and a negative C3d DAT, consistent with warm autoimmune hemolytic anemia (Table 1).

**Table 1:** Hematologic and Hemolysis Laboratory Evaluation During Hospitalization Laboratory findings demonstrated severe hemolytic anemia with reticulocytosis, hyperbilirubinemia, elevated LDH, reduced haptoglobin, and positive IgG DAT, supporting the diagnosis of warm autoimmune hemolytic anemia. LDH: lactate dehydrogenase, DAT: direct antiglobulin test

Parameter	Admission	Hospital day 7 (discharge)	Reference range
Hemoglobin (g/dL)	5.1	9.4	13.5-17.5
Reticulocyte %	41.3	-	0.5-2.5
LDH (U/L)	556	453	140-280
Haptoglobin (mg/dL)	<8	<8	30-200
Total bilirubin (mg/dL)	8.3	3.9	0.2-1.2
Indirect bilirubin (mg/dL)	6.7	3.1	0.2-0.8
Direct bilirubin (mg/dL)	1.6	0.8	0.0-0.3
Direct antiglobulin test	IgG positive	-	Negative
C3d DAT	Negative	-	Negative

**Figure 1:**
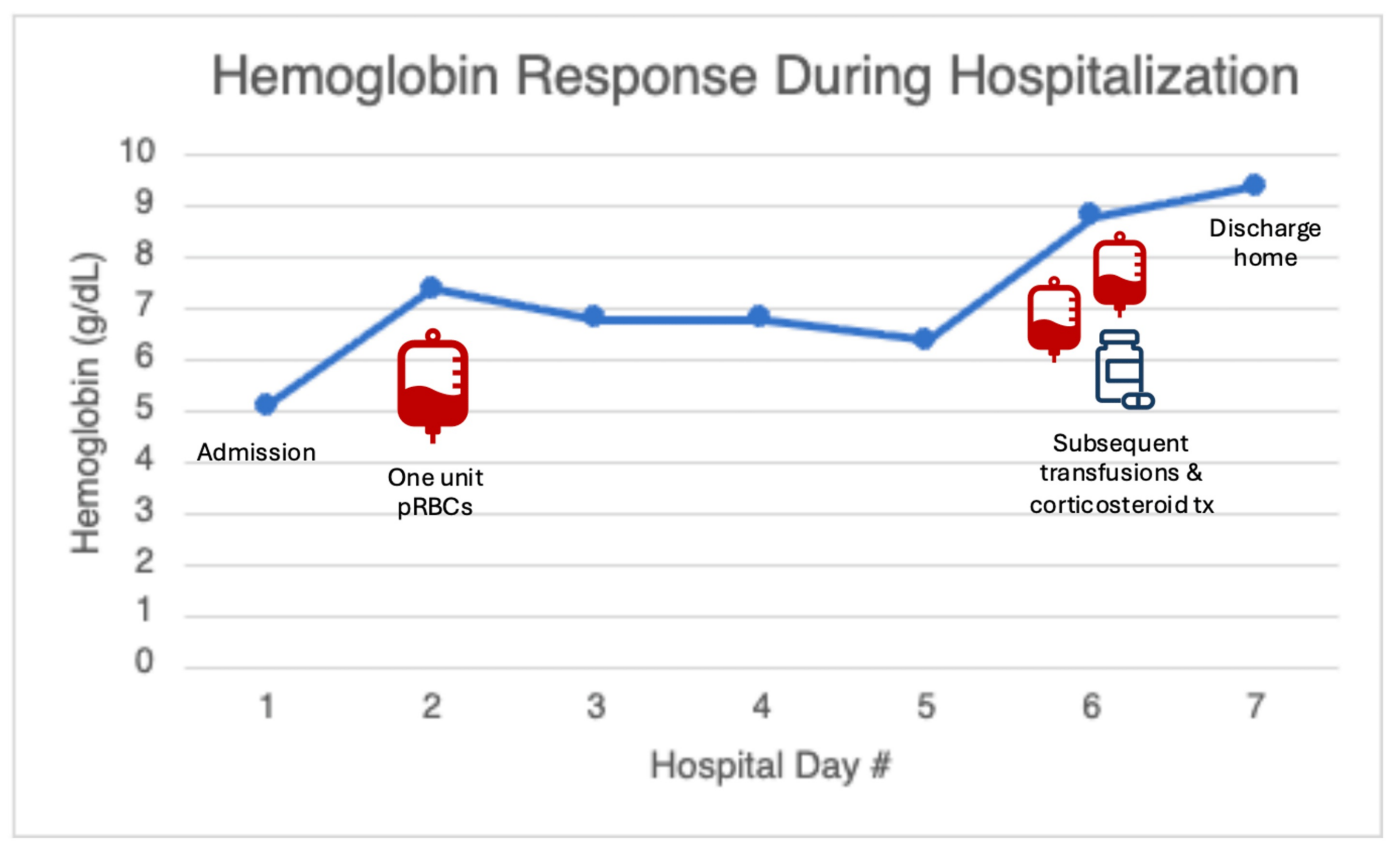
Clinical Timeline and Hemoglobin Response During Hospitalization Clinical course demonstrating severe anemia on admission with transient response to packed red blood cell transfusion (hospital days 1-2), recurrent decline due to ongoing hemolysis, and stabilization following initiation of corticosteroid therapy (day 3), followed by new-onset atrial flutter (days 4-5). Created in Microsoft PowerPoint; no AI tools used

On hospital day 1, the patient received one unit of packed red blood cells (pRBCs) with a transient improvement in hemoglobin. On hospital day 2, hemoglobin declined again due to ongoing hemolysis, necessitating two additional units of pRBCs. On hospital day 3, high-dose corticosteroid therapy was initiated, resulting in hematologic improvement and stabilization (Figure 1). On multiple occasions during hospitalization, when hemoglobin levels fell below 7 g/dL, transfusion was deferred as the patient remained hemodynamically stable and asymptomatic due to concern for exacerbating hemolysis and delayed hemolytic transfusion reactions.

Transfusion decisions were guided by clinical status rather than hemoglobin level alone, with avoidance when possible due to concern for exacerbating hemolysis, alloimmunization, and delayed hemolytic transfusion reactions.

On hospital day 4, the patient developed tachycardia and was found to have new-onset atrial flutter. This occurred in the setting of severe anemia and active hemolysis, which may have contributed to myocardial stress and a high-output physiological state. His CHA₂DS₂-VASc score of 4 indicated moderate-to-high thromboembolic risk; however, anticoagulation was deferred because of profound anemia, recent transfusion requirement, and concern for bleeding risk. Electrical cardioversion was also deferred because the duration of atrial flutter was unclear, and anticoagulation could not be safely initiated at that time. Rate control was achieved with metoprolol succinate 25 mg daily. Outpatient rhythm monitoring (e.g., Holter monitoring) and cardiology follow-up were recommended after hematologic stabilization. One week after discharge, follow-up laboratory studies demonstrated persistent hemolytic anemia, with hemoglobin of 8.2 g/dL, macrocytosis (mean corpuscular volume (MCV): 118 fL), elevated LDH (391 U/L), undetectable haptoglobin (<8 mg/dL), total bilirubin of 2.8 mg/dL, and reticulocytosis (10.5%).

## Discussion

This case illustrates the diagnostic and therapeutic challenges of warm AIHA in elderly patients with preexisting autoimmune cytopenias. In this patient, management of warm AIHA is complicated by preexisting comorbidities such as ITP and the concurrent discovery of atrial flutter, limiting the use of anticoagulation in the presence of hypercoagulable risk factors, including type 2 diabetes mellitus, hyperlipidemia, and advanced age. The sequential occurrence of ITP and AIHA fulfills diagnostic criteria for Evans syndrome [7], reflecting chronic immune dysregulation and predisposing patients to recurrent cytopenias. It is possible that the patient’s prior episode of anemia represented an earlier manifestation of Evans syndrome; however, the lack of hemolysis evaluation at that time limits definitive classification. Although Evans syndrome is more commonly described in younger populations, its presentation in older adults is often associated with a more refractory disease course, higher relapse rates, and increased morbidity due to comorbid conditions and treatment-related complications. In elderly patients, age-related immune dysregulation and impaired regulatory immune function may further contribute to loss of self-tolerance, while cumulative immunosuppression increases susceptibility to infection and limits therapeutic options. Prior corticosteroid exposure, immune senescence, and chronic immune activation may contribute to disease evolution in this population [6,7].

Warm AIHA is typically mediated by IgG autoantibodies directed against red blood cell membrane antigens, leading to splenic macrophage-mediated extravascular hemolysis [6,7]. This mechanism is confirmed by the positive IgG direct antiglobulin test (DAT). In older adults, the physiological decline in bone marrow reserve and reduced erythropoietin responsiveness can result in profound anemia due to an impaired compensatory reticulocyte response [5]. Erythropoietin therapy was not initiated because the patient demonstrated marked reticulocytosis, suggesting a robust preserved marrow response despite advanced age.

Management was guided by established recommendations for warm AIHA, including corticosteroids as first-line therapy. Although corticosteroid therapy posed a risk in this patient due to type 2 diabetes mellitus, it was selected because of the severity of hemolysis and need for rapid disease control. Glucose monitoring and outpatient adjustment of diabetic therapy were planned during corticosteroid treatment.

While corticosteroids remain first-line therapy [6], long-term use has numerous systemic adverse effects. The hyperglycemic effect can induce or worsen diabetes mellitus, inhibition of osteoblast activity can lead to osteoporosis, and suppression of the immune system increases the risk for infections. These side effects are particularly worrisome for the patient described in this case, because age alone predisposes him to a higher risk of metabolic and bone disease. Additionally, his comorbidities and age-related immune senescence weaken his immune system’s ability to fight off infection. Close monitoring for side effects and drug efficacy is imperative while elderly patients, especially those with several comorbidities, are being treated with corticosteroids [8].

Second-line therapies for warm AIHA include rituximab, intravenous immunoglobulin (IVIG), and other immunomodulatory agents, which may be considered in refractory disease [4,8]. Splenectomy is generally reserved as a last-line option, particularly in older patients, due to increased operative risk. Given this patient’s severe anemia requiring transfusion and history of autoimmune cytopenia, close follow-up is warranted to monitor for relapse or treatment resistance. In the setting of diabetes and a prior favorable response to rituximab for ITP, earlier consideration of rituximab may be appropriate if steroid toxicity develops or hemolysis recurs. IVIG may also be considered in cases requiring rapid immunomodulation or when there is an inadequate response to corticosteroids.

This patient’s concurrent development of atrial flutter further complicated management. Severe anemia induces a high-output state and myocardial stress [8], and the need to balance thrombotic risk with bleeding risk required multidisciplinary collaboration between hematology, cardiology, and internal medicine. Given the high bleeding risk associated with severe anemia, anticoagulation was deferred despite an elevated CHA₂DS₂-VASc score, representing a patient-specific deviation from standard atrial flutter management, and metoprolol was appropriately chosen for rate control. This case underscores the importance of individualized treatment strategies in geriatric patients with overlapping autoimmune and cardiovascular conditions. Early recognition of hemolysis and careful coordination of transfusion support, immunosuppressive therapy, and cardiovascular management are essential to minimizing morbidity in this population.

This case report has several limitations. First, the patient’s prior episode of anemia was not fully evaluated for hemolysis, limiting our ability to determine whether AIHA was already developing at that time. Second, although complement-specific testing with C3d DAT was performed, additional autoimmune evaluation was limited. Finally, because this is a single case, management decisions may not be generalizable to all elderly patients with warm AIHA or Evans syndrome.

## Conclusions

This case highlights the therapeutic complexity of warm AIHA in an elderly patient with prior ITP, diabetes mellitus, and new-onset atrial flutter. The key lesson is that management must be individualized when guideline-based therapy creates competing risks, such as corticosteroid toxicity in diabetes or anticoagulation in profound anemia. Early recognition of hemolysis, careful transfusion support, close monitoring for steroid complications, and multidisciplinary coordination are essential. Future cases may benefit from earlier consideration of steroid-sparing therapy when comorbidities increase the risk of prolonged corticosteroid exposure.
